# Suspected encephalitis in adults

**DOI:** 10.1136/pn-2024-004299

**Published:** 2026-04-29

**Authors:** Abdusshakur Muhammad Auwal, Rachael Matthews, Callum Cook, Brendan Sargent, Ava Easton, Stephen T J Ray, Mark Alexander Ellul, Benedict D Michael

**Affiliations:** 1 Institute of Infection, Veterinary and Ecological Science, University of Liverpool, Liverpool, UK; 2 Department of Neurology, The Walton Centre NHS Foundation Trust, Liverpool, UK; 3 Liverpool Interdisciplinary Neuroscience Centre, Liverpool, UK; 4 Liverpool University Hospitals NHS Foundation Trust, Liverpool, UK; 5 Autoimmune Psychosis Group, Department of Psychiatry, University of Oxford, Oxford, UK; 6 Encephalitis International, Malton, UK; 7 Department of Paediatrics, University of Oxford Oxford Vaccine Group, Oxford, UK

**Keywords:** CLINICAL NEUROLOGY, INFECTIOUS DISEASES, PSYCHIATRY

## Abstract

‘Query encephalitis’ is one of the most common reasons for inpatient neurology referral in the context of an acutely confused patient. Growing evidence suggests that time to treatment is a key determinant of outcome in both infectious and autoimmune encephalitis; hence, these two causes should be considered simultaneously at presentation. However, under-recognition and the existence of several mimics make a rapid diagnosis of encephalitis challenging. Appreciation of clinical syndromes can guide aetiological investigation and consequent treatment. In this article, we discuss clinical phenotypes associated with both infectious and autoimmune encephalitis, as well as a systematic approach to their investigation and up-to-date treatment strategies. We also highlight ongoing areas of research, such as metagenomics and therapeutic trials.

## Introduction

 Encephalitis is inflammation of the brain parenchyma, which is often due to an infection or an autoimmune process. It presents as a subacute alteration in mental state, with additional features such as fever, seizures or focal neurological deficits, depending on the underlying cause. Septic encephalopathy, on the one hand (table 1), may present similarly with fever, alteration in mental status and seizures, especially in the elderly who have a reduced cognitive reserve, but these patients usually have an alternative source of infection. The incidence of encephalitis is variable for several reasons, such as microbe distribution, but is around 4–12.6/100 000 people globally.^1–3^ While herpes simplex virus (HSV) encephalitis is the most common form of acute sporadic infectious encephalitis worldwide, endemic forms of infectious encephalitis are often confined to specific regions, such that geographical location can help to narrow down infectious causes.^3^ Moreover, being immunocompromised increases vulnerability to opportunistic pathogens. In addition to these established infectious causes, autoimmune causes of encephalitis have been increasingly recognised over the last two decades. Most cases of autoimmune encephalitis are associated with antibodies against neuronal surface epitopes (their location makes them more susceptible to immune therapy) and less commonly to intracellular antigens (which may require broader spectrum chemotherapeutic agents due to T-cells potentially driving disease pathogenesis) (figure 1, tables 2 and 3).^4^ Characterisation of the clinical phenotypes associated with these antibody-mediated disorders allows neurologists to identify patients who may respond to immune therapy. Early recognition and treatment of encephalitis is essential, as this significantly reduces both morbidity and mortality.^4^ In this article, we describe the clinical syndromes of the main causes of encephalitis in the UK, the diagnostic process and up-to-date management strategies since the 2012 National Encephalitis Guidelines.

**Table 1:** Differential diagnoses of encephalitis^87 88^

Category	Conditions to consider	Description	Investigations to consider
Infection	Sepsis	Encephalopathy is very common in patients with sepsis. Neuroimaging is usually normal (although it may show some ischaemic changes) and CSF is usually normal, although it may show raised protein.	Identification of alternative (non-CNS) focus of infection.
Toxic/metabolic	Toxic syndromes	Toxic syndromes can lead to seizures, changes in mental state and fevers. Examples include: neuroleptic malignant syndrome, serotonin syndrome, anticholinergic overdose, sympathomimetic overdose or toxicity from illicit drugs. Metabolic encephalopathy may result from uraemia, hepatic failure or inherited metabolic disorders.	History of use of culprit medications and clinical features. Asterixis (negative myoclonus) may be present in metabolic encephalopathies.^89^ It is largely a clinical diagnosis, but may be aided by blood tests, for example, showing renal or hepatic impairment, raised plasma ammonia or specific biochemical signatures of inherited disorders.^90^
	Hypoglycaemia, hyponatraemia or other electrolyte disturbances	May present with confusion, reduced level of consciousness and seizures. Should resolve with appropriate treatment	Low glucose (hypoglycaemia) or low sodium on laboratory testing.
Vasculitis	Primary CNS vasculitis	Rare, but if confined to CNS may not have usual systemic symptoms associated with vasculitis—joint pains, vasculitic rash, fever, malaise/anorexia	Presence of auto-antibodies specific for the condition, for example, ANA or dsDNA (SLE),^91^ cryoglobulinaemia)^92^
	Secondary CNS vasculitis	Most systemic vasculitides can progress to involve the CNS. Likely to have systemic symptoms.	Raised inflammatory markers, tissue biopsy.
	Secondary to inflammatory condition	Vasculitis may occur as part of another autoimmune or inflammatory condition—SLE, Sjögren’s syndrome, cryoglobulinaemia, rheumatoid arthritis.	Presence of auto-antibodies specific for the condition, for example, ANA or dsDNA (SLE)^91^ anti-Ro/anti-La (Sjögren’s syndrome)^93^ serum cryoglobulins (cryoglobulinaemia)^92^
Inflammatory/immunological	GFAP astrocytopathy	It can be easily confused with infectious encephalomyelitis, especially in patients presenting with triad of fever, headache and altered consciousness in addition to leptomeningeal enhancement, hence a worthy differential when initial investigations do not support infection	Positive GFAP– IgG antibodies in the CSF are diagnostic. Anti-NMDAR, anti-AQP4 and anti-MOG may coexist with these. Imaging to rule out associated malignancies such as ovarian teratoma, adenocarcinoma and glioma.^94^
	Neuromyelitis optica syndromes	Typically present as optic neuritis and longitudinal myelitis. Can less commonly present as intractable cough and hiccoughs, narcolepsy or even encephalopathy and seizures.^87^	Positive aquaporin-4 antibodies with positive MRI findings.^18^
	Haemophagocytic lymphohistiocytosis (HLH)	Inflammation secondary to immune activation, normally involving macrophages. Around one third of people have neurological involvement. Triggered by infection, rheumatological conditions or malignancy (especially lymphoma) Neurological symptoms are typically preceded by weeks of systemic symptoms.	Common laboratory findings: cytopenia (usually multiple cell lines), hypertriglyceridaemia, low fibrinogen, high ferritin (>500 ng/mL) and abnormal liver function (H-score can be used to estimate the probability of HLH in suspected individuals).^95^ Molecular diagnosis and bone marrow biopsy can be considered by a specialist.^96^
Rheumatological disorders	Systemic lupus erythematosus (SLE)	Rarely, SLE can have neuropsychiatric complications involving seizures, encephalopathy and psychosis. Pathophysiology is unclear, may be secondary to vasculitis, autoimmunity, infection or toxic side effects of treatment.	Presence of autoantibodies associated with SLE, including ANA or dsDNA ^91^
	Neurosarcoidosis	5%–10% of those with sarcoidosis develop neurosarcoidosis, but this can also develop in the absence of systemic sarcoidosis.	Serum and CSF ACE have poor sensitivity and specificity, especially if sarcoidosis is isolated to CNS. Non-specific inflammatory change on CSF analysis. MRI changes. Tissue biopsy if feasible.
Neoplastic	Temporal lobe tumours	Metastatic or primary tumours may present in a similar way to encephalitis. Repeat imaging showing static changes is helpful as other conditions such as HSV-1 encephalitis will show dynamic changes in the temporal lobes. Low-grade glioma, in particular, can mimic CNS infection.	Repeat neuroimaging showing static changes only, as opposed to evolving changes likely to be observed in true encephalitis. Definitive diagnosis requires histopathology.
	Intravascular large cell lymphomas	Rare subtype of large cell lymphoma often has CNS and cutaneous manifestations. B-symptoms will likely be present.	Common laboratory findings include raised LDH and B-2 microglobulin and cytopenias (anaemia, thrombocytopenia, leucopenia). Tissue required for definitive diagnosis.^67^
Others	Stroke-like migraine attacks after cranial radiation	Headaches and seizures which can occur years after cranial irradiation.	History of cranial radiation. MRI shows typical features of unilateral (usually the previously irradiated area) cortical oedema, not associated with vascular territories.^97^
	Syndrome of transient headache and neurological deficits with cerebrospinal fluid lymphocytosis	Self-limiting condition that may mimic encephalitis	Lumbar puncture shows lymphocytosis, and opening pressure may be raised.^98^
	Mitochondrial encephalomyopathies	Multiple disorders, one that most closely mimics encephalitis is MELAS (mitochondrial encephalopathy with lactic acidosis and stroke-like episodes)	Lactic acidosis. Neuroimaging may show stroke-like ischaemic lesions that do not follow vascular territories. Genetic testing.^99^
	Creutzfeldt-Jakob disease (CJD)	May mimic encephalitis, particularly the subacute course of autoimmune or limbic encephalitis.	EEG, MRI and CSF findings may indicate CJD, but empirical corticosteroids may be considered if the diagnosis is unclear.^100^

ANA, Anti-nuclear antibody; CNS, Central Nervous System; CSF, cerebrospinal fluid; dsDNA, Double-stranded DNA (deoxyribonucleic acid); EEG, electroencephalogram; HSV, herpes simplex virus; LDH, Lactate deyhdrogenase; MOG, myelin oligodendrocyte glycoprotein; NMDAR, N-methyl D aspartate receptor.

**Figure 1:**
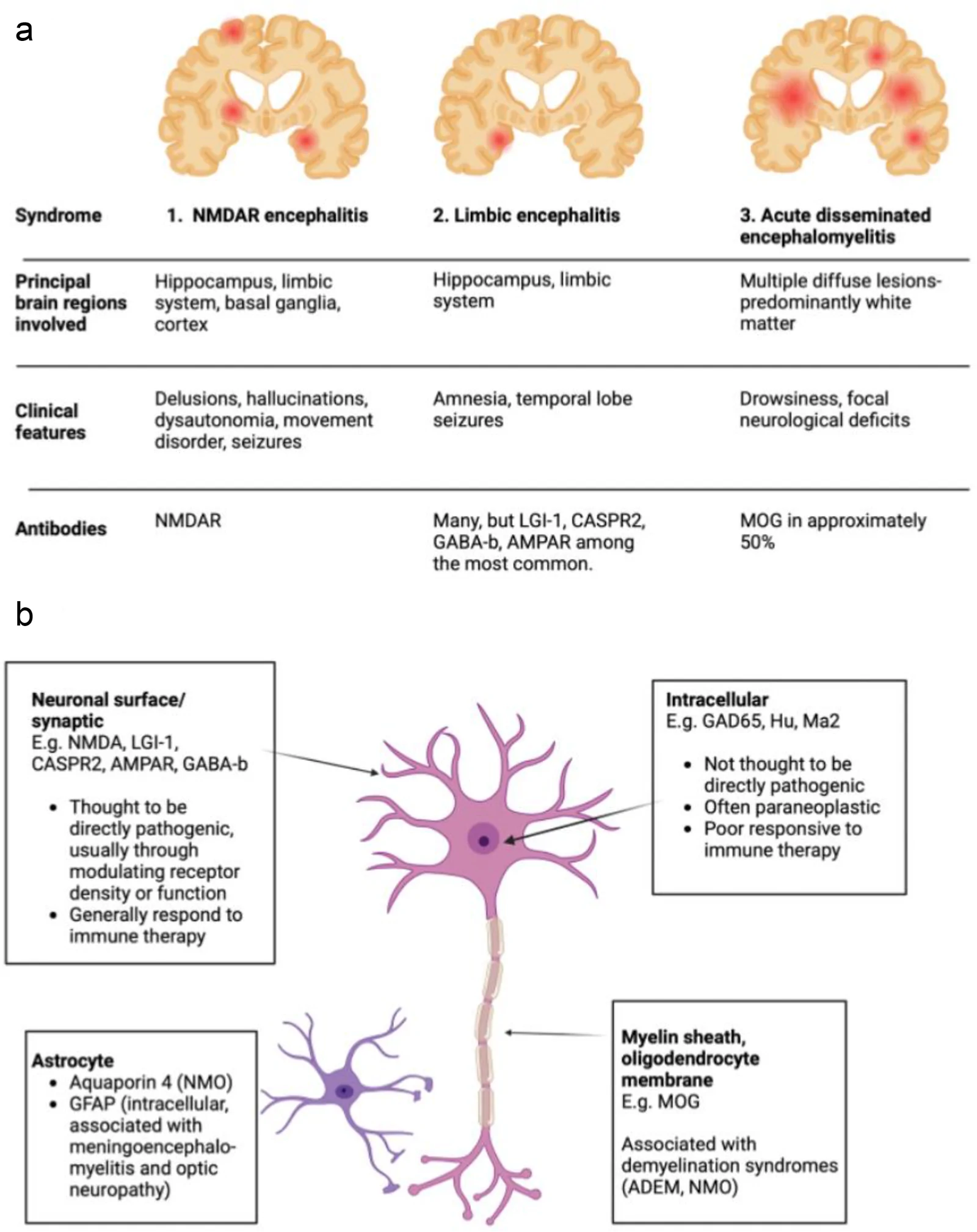
Clinicopathological conception of autoimmune encephalitis. (a) Key clinical syndromes of autoimmune encephalitis, main brain regions affected, clinical features and examples of associated antibodies. ‘Lumping’ syndromes can help to guide diagnostic strategies, although ‘splitting’ into individual antibodies is also informative, particularly for tumour associations and relapse risk. (b) Neuroglial antibody targets. Antibodies targeting neuronal surface/synaptic proteins can access and modulate their targets and generally respond to treatment. Intracellular antibodies cannot usually access their epitopes and are therefore probably epiphenomena of another pathogenic process, which may be T-cell driven. Other antibodies target the myelin sheath or glia. ADEM, acute disseminated encephalomyelitis; LGI-1, leucine-rich glioma inactivated 1; MOG, myelin oligodendrocyte glycoprotein; NMDAR, N-Methyl D aspartate receptor; NMO, neuromyelitis optica.

**Table 2:** Paraneoplastic/intracellular/glial antibodies^101^

Antibodies	Clinical presentation	Associated malignancy
MOG^102^	Acute disseminated encephalomyelitis	No known associated malignancy
Aquaporin-4	Encephalitis (mostly in children), neuromyelitis optica spectrum disorders	No known associated malignancy
Glycine-receptor	Progressive encephalomyelitis with rigidity and myoclonus, myelopathy, stiff person syndrome	~10% associated with thymoma or lymphoma
Dopamine-2-receptor	Basal ganglia encephalitis with gait disturbance, abnormal movement, psychiatric manifestations.	No known associated malignancy
ANNA1 (anti-Hu)	Limbic encephalitis, autonomic and peripheral neuropathies.	Small cell lung cancer (>90%)
ANNA2 (anti-Ri)	Brainstem encephalitis and cerebellar degeneration	Small cell lung cancer, breast and bladder cancers (>60%)
GAD-65^103^	Stiff-person syndrome, cerebellar ataxia, limbic encephalitis	No known associated malignancy
GFAP^104^	Subacute meningoencephalitis, cerebellar dysfunction, myelitis, peripheral nervous system involvement	Various malignancies (lymphomas, lung adenocarcinoma, ovarian teratoma)
mGluR5^105^	Limbic encephalitis with neuropsychiatric features	Hodgkin’s lymphoma
Ma-2^106^	Limbic encephalitis, diencephalon and brainstem dysfunction	Testicular germ cell tumours in >50% other cancers including non-SCLC form the rest

MOG, myelin oligodendrocyte glycoprotein; SCLC, small cell lung cancer.

**Table 3:** Neuronal surface and synaptic proteins antibodies, their syndromic presentation and association with malignancy^107^

Antibody	Clinical features	Associated malignancy
NMDA-receptor	Influenza-like prodrome, followed by behavioural change, impaired cognition and neuropsychiatric symptoms. Associated with dysautonomia, movement disorder, seizures, coma and central apnoea. Female to male predominance of 4:1.	Ovarian teratoma (37%) In older, male patients, other neoplasia may be present.^108^
LGI-1*	Faciobrachial dystonic seizures, behavioural change, impaired cognition, seizures. Associated hyponatraemia.	<10% associated with malignancy (breast, thyroid, lymphoma, thymoma)
CASPR2*	Most commonly affects older men. Associated with limbic encephalitis, peripheral nervous system hyperexcitability, autonomic dysfunction, neuropathic pain, cerebellar dysfunction. Male to female predominance of 9:1.	Thymoma (20%–50%), especially when LGI-1 antibodies are also present.
GABA-A	Encephalitis, seizures, common cause of status epilepticus.	Thymoma
GABA-B	Limbic encephalitis, ataxia, seizures, orolingual movements	Small cell lung cancer (50%) Neuroendocrine tumours
AMPA^109^	Sudden short-term memory loss, limbic encephalitis, psychiatric symptoms.	Around 62% had lung cancer or thymoma. Psychiatric symptoms at presentation possibly predict a risk of malignancy.
DPPX^47 110^	Classical triad of chronic diarrhoea and weight loss, impaired cognition and CNS hyperexcitability (myoclonus, tremors, hyperekplexia). Hallucinations and brain stem involvement can also occur.	~10% have B-cell lymphoma or leukaemia
IgLON5*^111^	Sleep disorders, bulbar syndrome, gait instability and cognitive impairment. Often overlaps with other neurodegenerative tauopathies.	No known association with malignancy

*These belong to the IgG4 subclass, do not activate complement, present subacutely and respond better to B-cell depleting therapies

LGI-1, leucine-rich glioma inactivated 1; NMDA, N-methyl D aspartate.

## Clinical syndromes and localisation

An acute or subacute onset of altered mental state, occurring from hours, days and occasionally weeks, is a defining historical feature of encephalitis (table 4).^5^ This alteration can be a subtle change in behaviour or personality, or as overt as amnesia, confusion or coma.^4 6^ Given that symptoms can fluctuate and the patient may have limited insight into their mental state, taking a collateral history from a close contact is critical.

**Table 4:** Criteria for defining encephalitic syndrome (modified from: Diagnostic Criteria for Encephalitis and Encephalopathy of Presumed Infectious or Autoimmune Aetiology)^5 29^

Major criterion (required)	Minor criteria (at least two required)
Subacute alteration in mental status (decreased consciousness, lethargy or behavioural change) or psychiatric symptoms, lasting greater than 24 hours with no identifiable alternative cause	Fever (≥38°C) in the 72 hours before or after presentation
	Generalised or partial seizures not due to pre-existing seizure disorder
	New onset of focal neurology
	CSF white blood cell pleocytosis (≥5/mm^3^)
	Appearances of acute abnormality on neuroimaging that is consistent with encephalitis

CSF, cerebrospinal fluid.

Testing for infectious causes should be guided by clinical features, including travel and vaccination history. Encephalitis syndromes can be localised to the brain regions most affected, through the presence of neurological deficits, seizure semiology or cognitive features, and certain syndromic clusters strongly suggest autoimmune aetiology (box 1).^7 8^ Clues in the history and examination may help localise the site of inflammation and provide insight into the underlying aetiology, while global inflammatory processes may present with coma, making it much more challenging to identify the underlying cause.^9–11^

Box 1 **Clinical features to help distinguish infectious from autoimmune cause^7^**

**Clinical features favouring an autoimmune cause:**

Recent coryzal illness or vaccination (particularly acute disseminated encephalomyelitis)

Neuropsychiatric symptoms: subacute behavioural change, impaired cognition, psychosis

Movement disorder, particularly with orofacial movements or athetoid limb movements, suggesting N-Methyl D aspartate receptor (NMDAR)-antibody encephalitis

Underlying neoplasm, for example, an ovarian teratoma (NMDA-receptor encephalitis), small cell lung cancer (gamma-aminobutyric acid-b)

Patient demographics: young female (NMDA receptor antibody encephalitis), older male (leucine-rich glioma inactivated 1 (LGI-1)/contactin-associated protein like 2 (CASPR-2) antibody-mediated encephalitides)

Disease specific: orofacial dyskinesia and psychosis (NMDA-R antibody encephalitis), faciobrachial dystonic seizures, new-onset refractory status epilepticus, hyponatraemia (LGI-1 antibody encephalitis), peripheral nervous system hyperexcitability, autonomic and cerebellar dysfunction, neuropathic pain (CASPR-2 antibody encephalitis)

**Clinical features favouring an infective cause:**

Prodrome of coryzal illness

Fever (which may be fluctuant)

Unwell contacts

Rash, for example, measles (viral exanthem, begins around neck and ears, spreads to trunk and extremities), parvovirus (erythema infectiosum), human herpesvirus type 6B (roseola infantum)

Environmental exposure: proximity to animals, sexual contacts (syphilis), travel history (endemic areas for Japanese encephalitis, West Nile virus)

Immunocompromise: HIV/AIDS, immunosuppressive medications, disorders of immune system.

Déjà vu and olfactory hallucinations, together with an amnestic syndrome, suggest medial temporal lobe and limbic system involvement. An acute onset, over hours to days, suggests possible HSV−1 encephalitis, while a subacute onset, over days to weeks, suggests autoimmune limbic encephalitis.^12^ Specifically, leucine-rich glioma (LGI)−1 antibody-mediated encephalitis is also often associated with faciobrachial dystonic seizures, which are pathognomonic,^13^ and contactin-associated protein (CASPR)−2 antibody encephalitis is often described as the triad of Central Nervous System (CNS) dysfunction, autonomic disturbance and peripheral nerve hyperexcitability, termed Morvan’s syndrome.^14^

Encephalitis due to N-methyl D aspartate receptor (NMDAR) antibody encephalitis presents with a well-characterised clinical syndrome including early psychiatric features such as delusions and hallucinations, movement disorder (eg, orofacial dyskinesia), dysautonomia, seizures and coma.^10–12^ Since the psychiatric features, aggression or irritability can be prominent, it can be challenging to distinguish from an acute psychosis, with negative and positive symptoms, and occasionally catatonia.^15^ The movement disorder of NMDAR-antibody encephalitis is heterogeneous, but often involves orofacial movements (sometimes referred to as *dyskinesia*) along with athetotic or choreiform limb movements.^16^ NMDAR-antibody encephalitis is about four times more common in females than males, who frequently have ovarian teratoma, although male patients (usually without tumour) can also be affected.^15 17^

Brainstem encephalitis (rhombencephalitis) may present with cranial nerve palsies, ataxia or hemiparesis and sometimes with preserved behaviour and consciousness.^4^ Area postrema involvement within the medulla can present with intractable vomiting and hiccoughs.^18^ Possible causes include Listeria, Brucella, arthropodborne viruses and enterovirus infection. Some enterovirus strains affecting the brainstem (eg, EV71) can cause dysautonomia syndrome, presenting with features such as labile blood pressure.^19^

Various autoimmune encephalitides may also involve the brainstem. These include: Bickerstaff’s brainstem encephalitis, defined by the triad of altered consciousness/hypersomnolence, ophthalmoplegia and ataxia with positive anti-GQ1b antibodies and often preceded by an infectious trigger,^20^ myelin oligodendrocyte glycoprotein (MOG)-antibody-associated disease (MOGAD)—described below, anti-IgLON5 disease (table 3) and GFAP astrocytopathy (table 1). Some paraneoplastic syndromes, such as anti-Hu, anti-Ma2, anti-Ri (table 2) and Kelch-like protein-11 (KLHL11), and Leucine Zipper 4 (LUZP4) antibodies, also cause brain stem dysfunction. LUZP4 and KLHL11 are both associated with germ cell tumours, the latter being poorly responsive to immunotherapy, and the coexistence of both is associated with a poor outcome.^20^

**Table 5:** Interpretation of CSF findings^113 114^

CSF measurement	Viral	Non-viral infective cause	Autoimmune
Opening pressure	Normal/raised	Raised	Normal/raised
White cell count	Moderately raised (10–200 cells/µL^3^); may be normal in early infection	Raised, predominantly neutrophils	Raised, pleocytosis in most cases, but may be normal.
CSF:blood glucose ratio	Normal	Low	Normal
Protein	Mildly elevated (typically, 0.5–1.0 g/L)	Raised	Raised (especially in anti-NMDAR) May be normal
Xanthochromia	Possible in HSV-1 and VZV	Negative	Negative

CNS, Central Nervous System; CSF, cerebrospinal fluid; HSV, herpes simplex virus; NMDAR, N-methyl D aspartate receptor; VZV, varicella zoster virus.

A progressive history of rapid decline in consciousness, Parkinsonian tremors and other movement disorders suggests basal ganglia or thalamic involvement. This is most commonly due to arthropodborne viruses such as Japanese encephalitis virus, West Nile virus or Eastern equine virus.^21^ These pathogens, which may be found in returning travellers, together with enteroviruses, can also present with acute flaccid paralysis when the anterior horn cells of the spinal cord have been infected.^19 22^

Acute disseminated encephalomyelitis (ADEM) presents mostly in children and young adults with encephalopathy and multifocal neurological signs due to diffuse demyelination involving the white matter of the brain and spinal cord. There is frequently an antecedent infection or vaccination, and about half of cases have positive MOG antibodies.^23–25^ While post-infectious or para-infectious ADEM is mainly confined to the white matter, a multifocal encephalitis due to direct CNS infection (eg, with arboviruses or enterovirus) involves the grey matter.^26^ Additionally, MOGAD has heterogeneous presentations, including cerebral cortical encephalitis, characterised by cortical hyperintensity on FLAIR MRI.^27^ Rasmussen’s encephalitis is a rare progressive unilateral encephalitis that tends to present with hemiplegia, cognitive decline and refractory focal epilepsy.^28^

Infectious encephalitis is often accompanied by fever (which may be fluctuant), although fever may also occur in autoimmune encephalitis owing to autonomic dysregulation. Infectious causes may be suggested by unwell contacts, or rash such as a vesicular rash of shingles (varicella zoster virus, VZV), insect bites (tickborne encephalitis virus, arboviruses, Lyme), hand-foot-and-mouth (enteroviruses), purpuric rash (meningococcaemia), track marks in an intravenous drug user (HIV) or other causes (eg, measles, parvovirus or Human Herpes Virus type 6B (HHV-6B)). Additionally, infection is suggested in those with appropriate animal contacts (eg, cats with *Bartonella spp*), or exposure to a vector known to transmit infectious agents (eg, mosquitoes, ticks), travel to an endemic area and immunosuppression (eg, HIV/AIDS, chemotherapy or post-transplantation) (figures 2 and 3).

**Figure 2:**
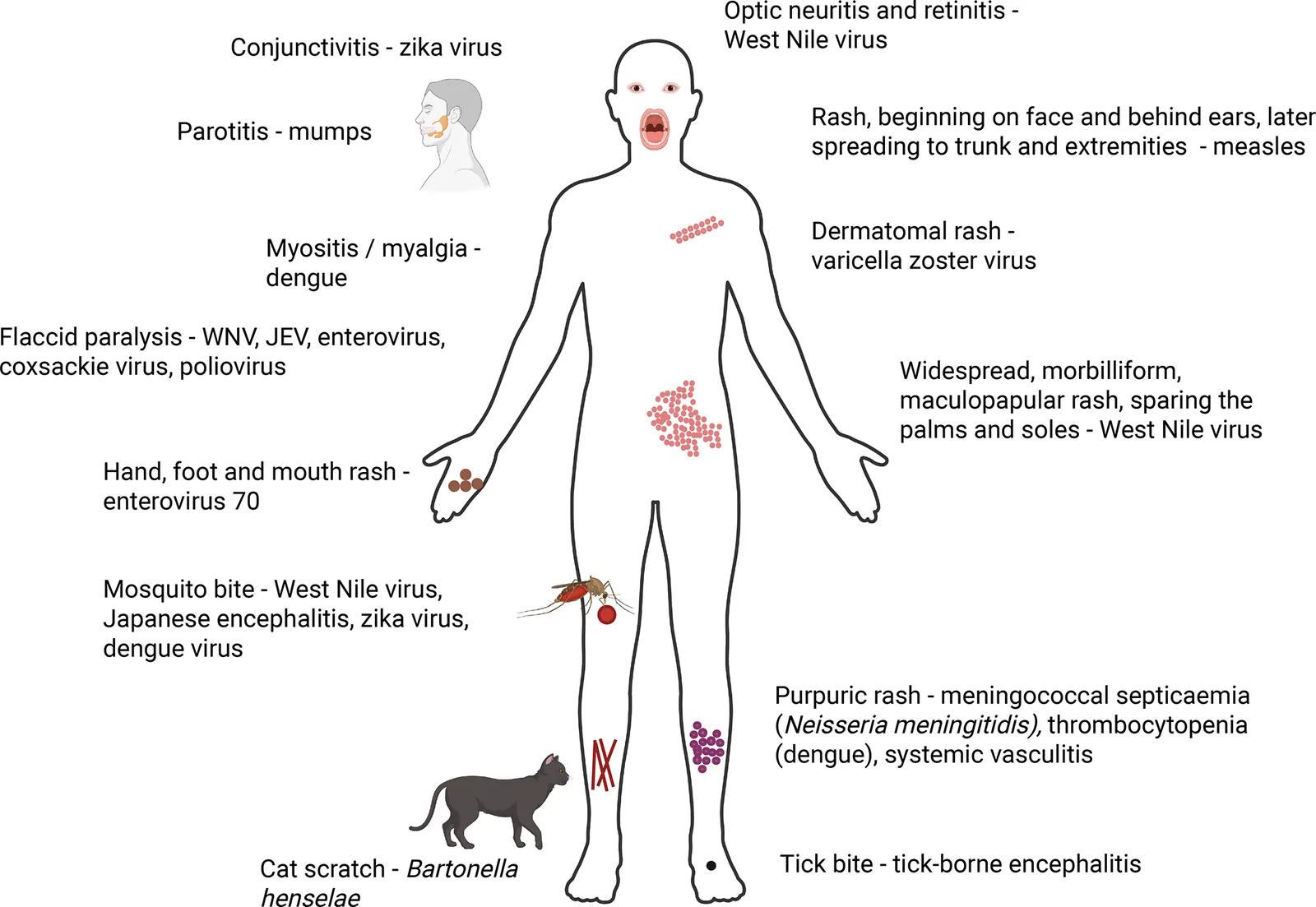
A diagram showing examination signs in immunocompetent patients, pointing towards an underlying cause. JEV, Japanese encephalitis virus; WNV, West Nile virus.

**Figure 3:**
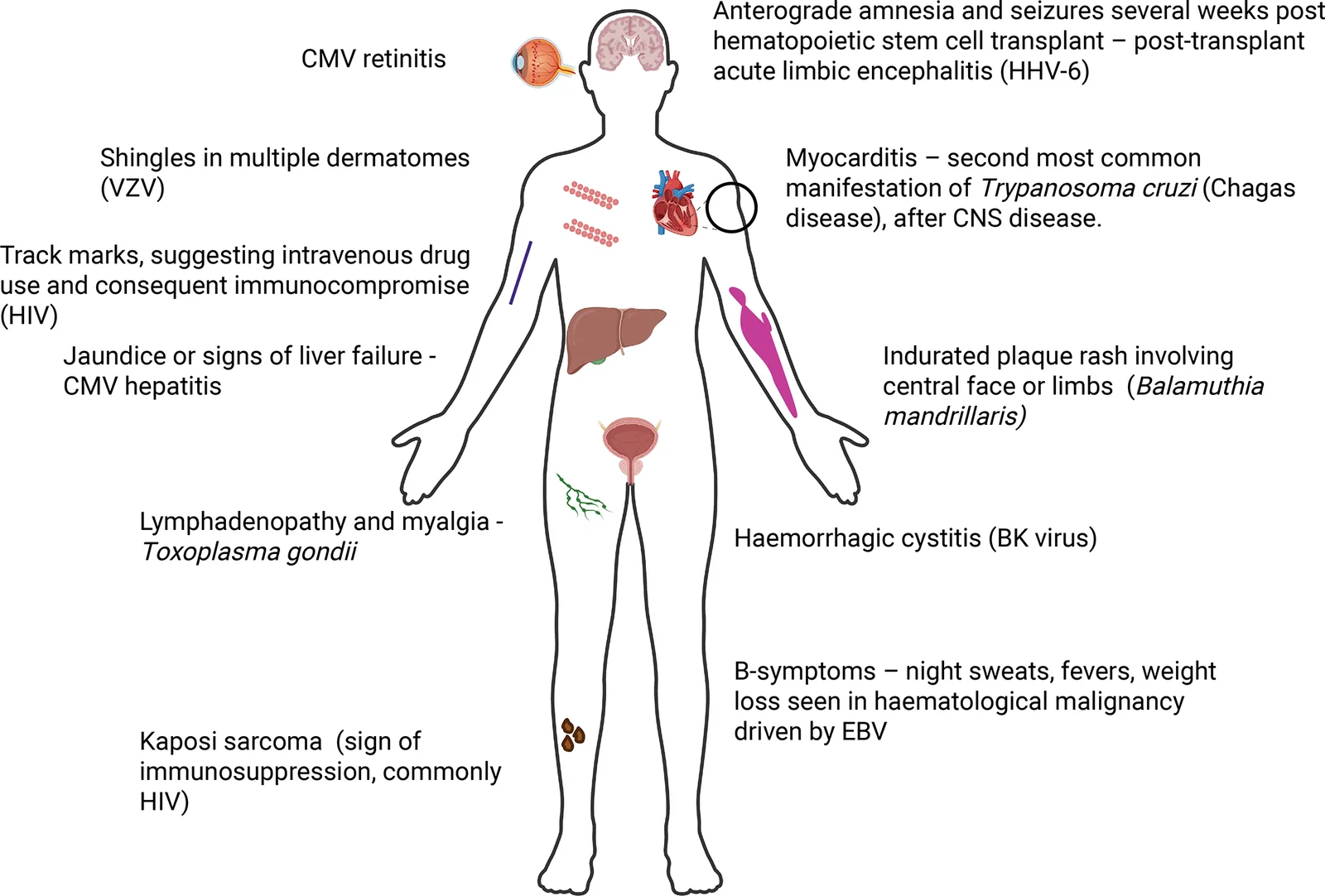
A diagram showing examination signs in immunocompromised patients pointing towards an underlying cause.^26 115–118^ CMV, cytomegalovirus; EBV, Epstein-Barr virus; HHV-6, human herpes virus; VZV, varicella zoster virus.

Consensus criteria were proposed for autoimmune encephalitis in 2016, based on categorising antibody-mediated diseases into recognisable clinical syndromes, including autoimmune limbic encephalitis, NMDAR-antibody encephalitis, ADEM and others.^29^ The antibody-negative criteria fall into two categories: ‘definite autoimmune limbic encephalitis’ and ‘autoantibody-negative but probable autoimmune encephalitis’, both of which require strict objective evidence of CNS inflammation and exclusion of alternative causes.

## Identifying the cause

Investigations are performed to confirm inflammation of the brain, identify the cause and to rule out complications. The differential diagnosis is vast; as such, clinicians should tailor investigations to the clinical context, and the results should iteratively guide additional investigations.

## Blood and cerebrospinal fluid analysis

A full blood count is often normal, but may show lymphopenia with viral infections, while eosinophilia mostly occurs in fungal and parasitic infections.^30^ Serum C reactive protein may be normal in encephalitis since the inflammation is typically confined to the CNS without peripheral involvement. Hyponatraemia secondary to syndrome of inappropriate antidiuresis (SIAD) or cerebral salt wasting can occur in infectious encephalitis, while hyponatraemia (mostly owing to SIAD) also occurs in autoimmune encephalitis (eg, LGI-1).^6^

All patients suspected of having CNS infection or inflammation should undergo HIV testing, as this can cause encephalitis directly at seroconversion or, in established infection and immunocompromise, can raise the possibility of opportunistic infection.^31^

Obtaining cerebrospinal fluid (CSF) via a lumbar puncture (LP) is imperative to the diagnosis of encephalitis. Unless there is a clinical contraindication, such as a history of bleeding diathesis, taking anticoagulant or antiplatelet medication, being immunocompromised or having clinical features suggesting brain shift causing raised ICP, there is usually no need to wait for a clotting test or brain imaging before performing an LP in a patient with suspected encephalitis.^32–34^

In addition to measuring the CSF opening pressure, initial CSF tests should include biochemistry (protein, glucose and oligoclonal bands—paired with analysis in serum), differential cell count and microbiology (Gram stain, culture and sensitivity) (table 5). A pleocytosis (≥5 cells) or elevated protein suggests neuroinflammation. In general, infection produces a significantly elevated white cell count with a predominance of lymphocytes (suggesting a viral cause) or neutrophils (suggesting a bacterial cause). However, neutrophils may predominate early in viral encephalitis.^35^ Red cells can be significantly raised in HSV-1 encephalitis or acute haemorrhagic necrotising encephalitis.^6^ Eosinophils may occur with helminthic, fungal or mycobacterial encephalitis.^4^ Autoimmune encephalitis may also show a modest lymphocytic pleocytosis.^36^ However, having an unremarkable CSF does not exclude encephalitis, as about a third of autoimmune encephalitides have a normal routine CSF analysis, as well as a proportion of those with viral encephalitis. In this case, LP should be repeated 48–72 hours later if there is ongoing suspicion of possible encephalitis.^6 36^

Traumatic LPs (defined as >1000 red cells in the CSF^37^) need to be interpreted carefully since the presence of blood can falsely elevate the CSF white cells and protein. Correction factors such as allowing two white cells in the CSF for every 1000 red cells and 0.0 l g/L of protein for every 1000 red cells have been suggested in the literature, although this approach needs to be applied with caution due to varying degrees of accuracy.^38–40^

CSF should be sent for a viral PCR panel (at least including HSV 1&2/VZV/enterovirus). The CSF can sometimes be PCR negative despite clinical features suggesting viral encephalitis, particularly if the LP is performed in the first few days of illness or after having started aciclovir. In this case, the LP should be repeated after 48 hours while continuing aciclovir in the interim. In cases where radiological appearance and clinical features suggest viral encephalitis, but the PCR is negative, intrathecal antibody testing (IgM and IgG) can be arranged through the reference laboratory at UKHSA (paired serum and CSF should be sent at least 10 days after symptoms onset).^41 42^ Additional tests are guided by the clinical context and initial CSF findings. For example, Epstein-Barr virus/cytomegalovirus (CMV)/HHV 6B & 7 PCR should be tested in the immunocompromised, adenovirus/rotavirus/influenza A&B in children, measles, mumps, chlamydia when indicated, and others such as rabies, West Nile virus, Japanese encephalitis virus and tickborne encephalitis depending on exposure.^6^ Serial blood and CSF cultures should be taken when suspecting *Listeria monocytogenes* encephalitis, MTB Xpert/RIF assay for *Mycobacterium tuberculosis* and antigen tests for fungal agents like *Cryptococcus spp* (CrAg).^43–45^

It is worth noting that due to the variable sensitivities of multiplex PCR (such as Biofire) especially for viruses, patients with a negative test despite a high clinical suspicion require a repeat PCR with a singleplex PCR.^46^

Patients with a suspected autoimmune cause should be tested for autoantibodies in both serum and CSF. Specificity for NMDAR antibodies in particular, and probably most others, is higher in CSF than serum. However, serum testing may be more sensitive, and CSF may be falsely negative in some cases, particularly for LGI-1 and MOG antibodies.^9 47^ Antibodies are most accurately tested by cell-based assay, as antibody binding depends on presentation of the epitope in its native conformation. Increasingly, laboratories offer fixed-cell-based assay panels, which have the advantage of testing the most common antibodies simultaneously; however, these should be used with caution in cases with a low pretest probability. False positive results can occur, particularly with low-titre CASPR2 antibodies.^48^ In the case of positive results without the expected clinical syndrome, or unexpected negative results, advanced assays available in research laboratories may provide clarity. Live cell-based assay may provide additional value above the fixed panels commonly offered in diagnostic laboratories, which can reduce non-specific binding to intracellular epitopes. Immunohistochemical tissue-based assays, or staining of primary neurones, may also be used as a confirmatory test to improve specificity or as a screening test for a larger range of antibodies, especially in ’seronegative’ cases.^49^ Antibodies against the voltage-gated potassium channel should no longer be tested, as they have a high false-positive rate and have no diagnostic value over testing LGI-1 and CASPR2 antibodies, which provide added specificity to the clinical syndrome, clinical relevance and associations with malignancy.^50^

## Neuroimaging

CT brain imaging is usually obtained in the acute setting due to availability, although it is much less sensitive than MR imaging in detecting encephalitis.^51^ MRI may show brain parenchymal inflammation and give clues to the possible cause (figures 4–6) based on the inflammation pattern and is important in excluding mimics. It is important to note that MR brain imaging can be normal or show non-specific changes, especially in autoimmune encephalitides.^52^

**Figure 4:**
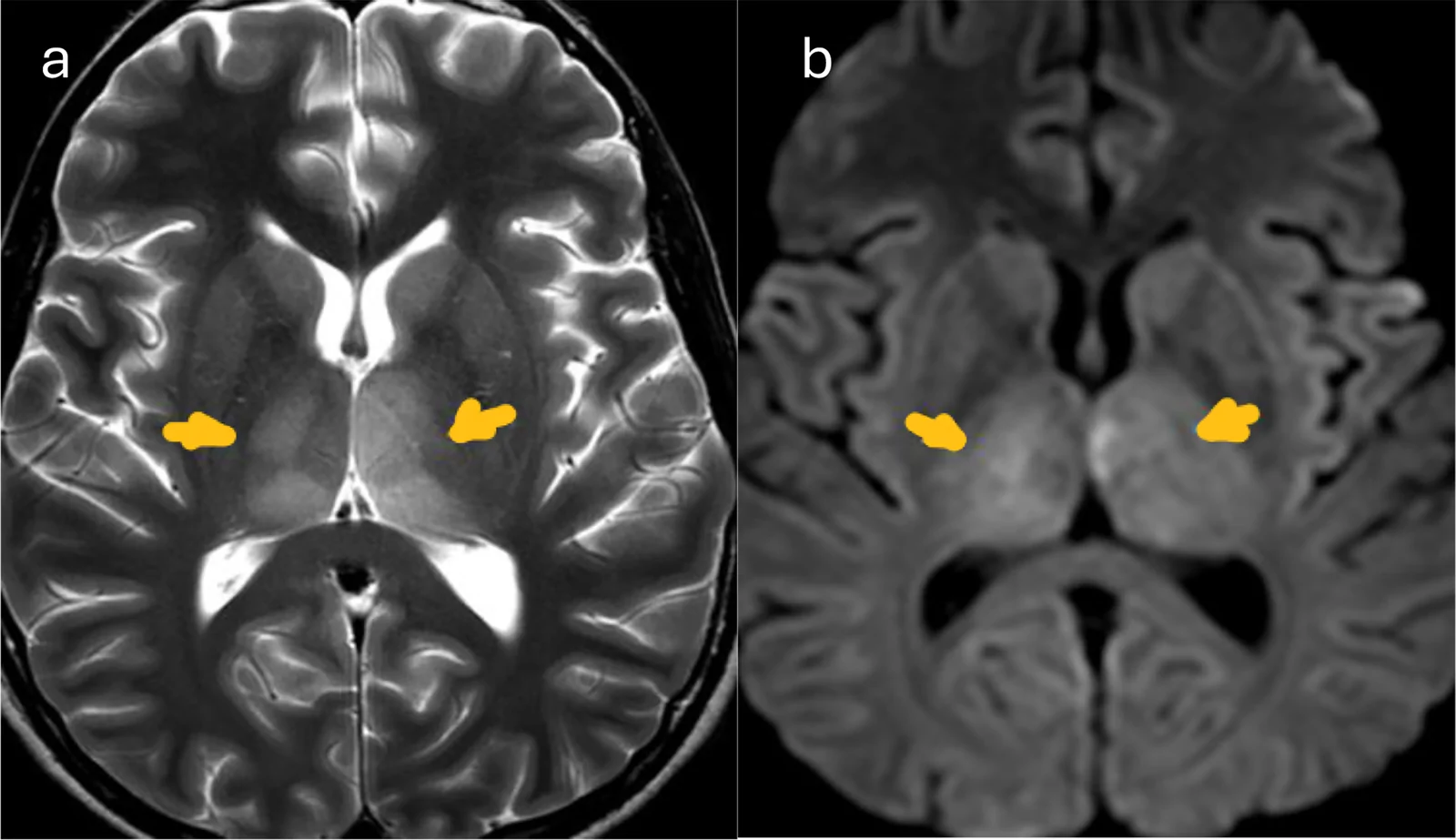
Images from a confirmed case of Japanese encephalitis showing (a) T2 hyperintensity involving bilateral thalami and midbrain and (b) cortical restricted diffusion in the left medial temporal lobe and patchy restricted diffusion in bilateral thalami.

**Figure 6:**
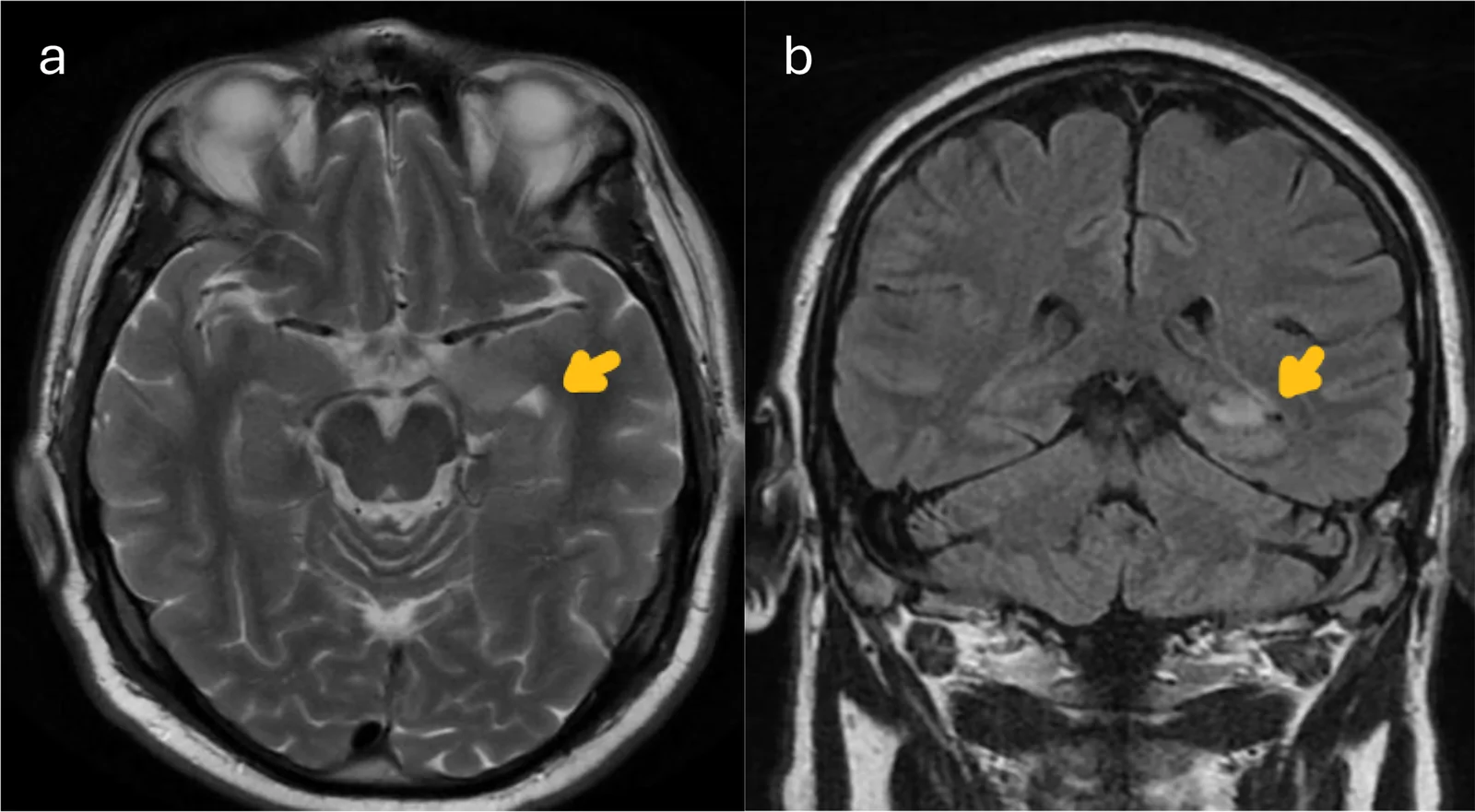
MR scan of brain a 55-year-old man with LGI-1 encephalitis (a) Axial T2 weighted and (b) Coronal T2 FLAIR, all showing high signal changes in the left temporal lobe. FLAIR, Fluid Attenuation Inversion Recovery; LGI-1, leucine-rich glioma inactivated 1.

**Figure 5:**
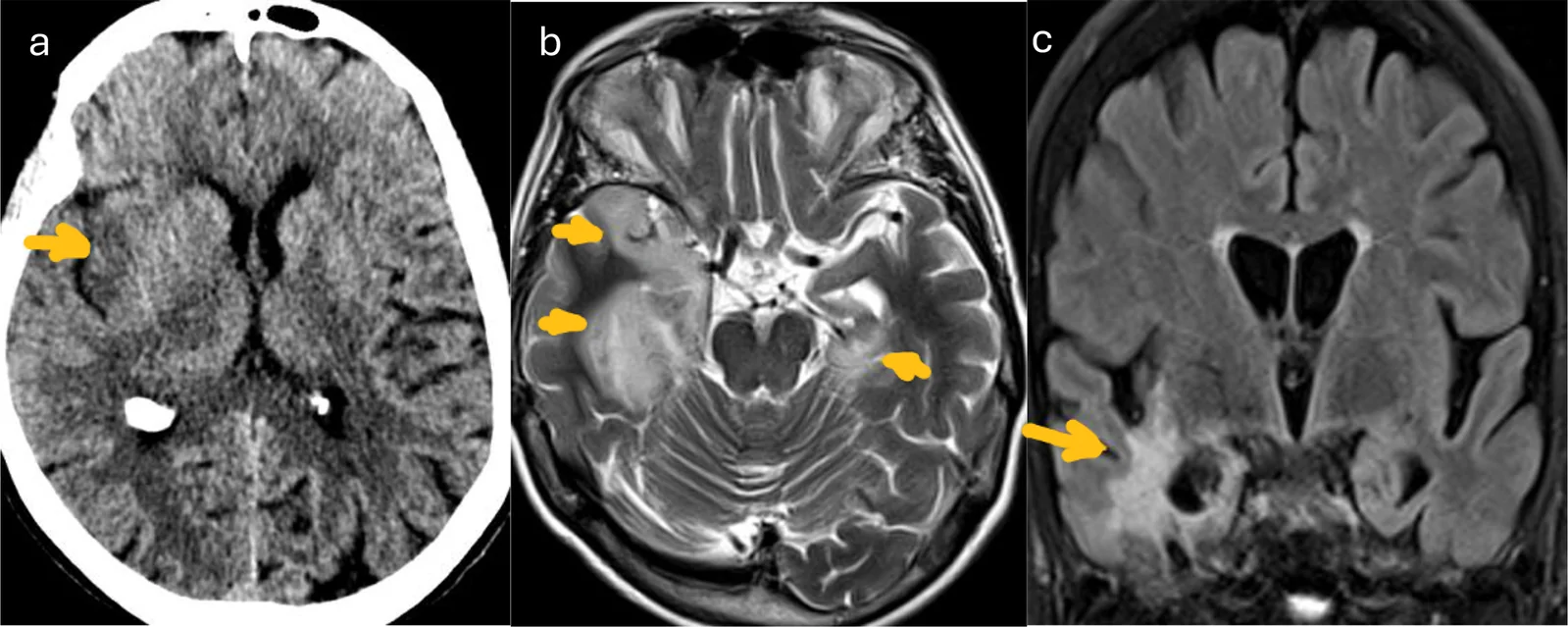
Imaging of a 72-year-old man presenting with acute confusion, later diagnosed with herpes simplex encephalitis. (**a**) CT scan of head showing cortical and subcortical white matter hypodensity affecting the anterior and medial right temporal lobe, right insula and left medial temporal lobe. (**b**) MR scan of brain showing asymmetric bilateral temporal lobe T2 hyperintensity with cortical restricted diffusion. (**c**) MR scan of brain 12 months later showing marked gliosis of the right antero-medial temporal lobe and right insula.

FDG positron emission tomography (PET-CT) of the brain has been used in those with a strong suspicion of encephalitis where the MRI is normal or antibody-negative patients with suspected autoimmune encephalitis, although this is not validated for clinical practice.^53 54^ Body PET-CT should also be performed as part of a malignancy screen in patients with autoantibodies with tumour associations (see table 2).

Single-photon emission CT and MR spectroscopy are not routinely used in clinical practice but are the subject of ongoing research.^55 56^

## Electroencephalogram

Electroencephalogram (EEG) is likely to show evidence of encephalopathy or identify non-convulsive or subtle motor status in the context of encephalitis.^51^ Occasional patients with NMDAR-antibody encephalitis show an ‘extreme delta brush’ pattern, which suggests an increased risk of a poor outcome.^57 58^

## Other investigations

Sites outside the CNS could be the potential source of pathogens and consequent brain inflammation either directly or indirectly. Therefore, consider PCR of respiratory samples for influenza, chlamydiae and SARS-CoV-2, of skin vesicles for VZV and enterovirus and rectal swabs for enterovirus. However, throat and rectal swabs can be positive in the absence of infection, due to the asymptomatic shedding of enteroviruses.^51^

A brain biopsy was widely used to diagnose encephalitis before the advent of CSF PCR. It is now typically indicated only in patients with evidence of brain inflammation of unexplained cause that is refractory to encephalitis treatment, or there is suspicion of another diagnosis such as CNS malignancy.^4 6 59^

Metagenomic sequencing is not yet widely available in clinical practice. It has the potential to identify novel or unexpected pathogens. However, it requires expertise to distinguish causative pathogens from contaminants or bystanders; it also requires confirmatory testing, and so presently is less sensitive than the conventional PCR. The approach is particularly promising in tissue (e.g. from brain biopsy) and in immunocompromised patients.^60 61^

## Management

The traditional approach to managing patients with suspected encephalitis is centred on treating for and excluding infectious encephalitis, before consideration of other causes, including autoimmune encephalitis, in sequential steps. However, in Western populations, the incidence of autoimmune encephalitis appears to be similar to that of infectious encephalitis, and the detection of antibodies is increasing annually.^62^ The mortality and morbidity of some forms of autoimmune encephalitis also approach or exceed that of viral encephalitis.^63^ It is therefore important to consider and investigate for autoimmune and infectious causes at the outset, with careful consideration of the tempo of the illness and the clinical syndrome (figure 7).

**Figure 7:**
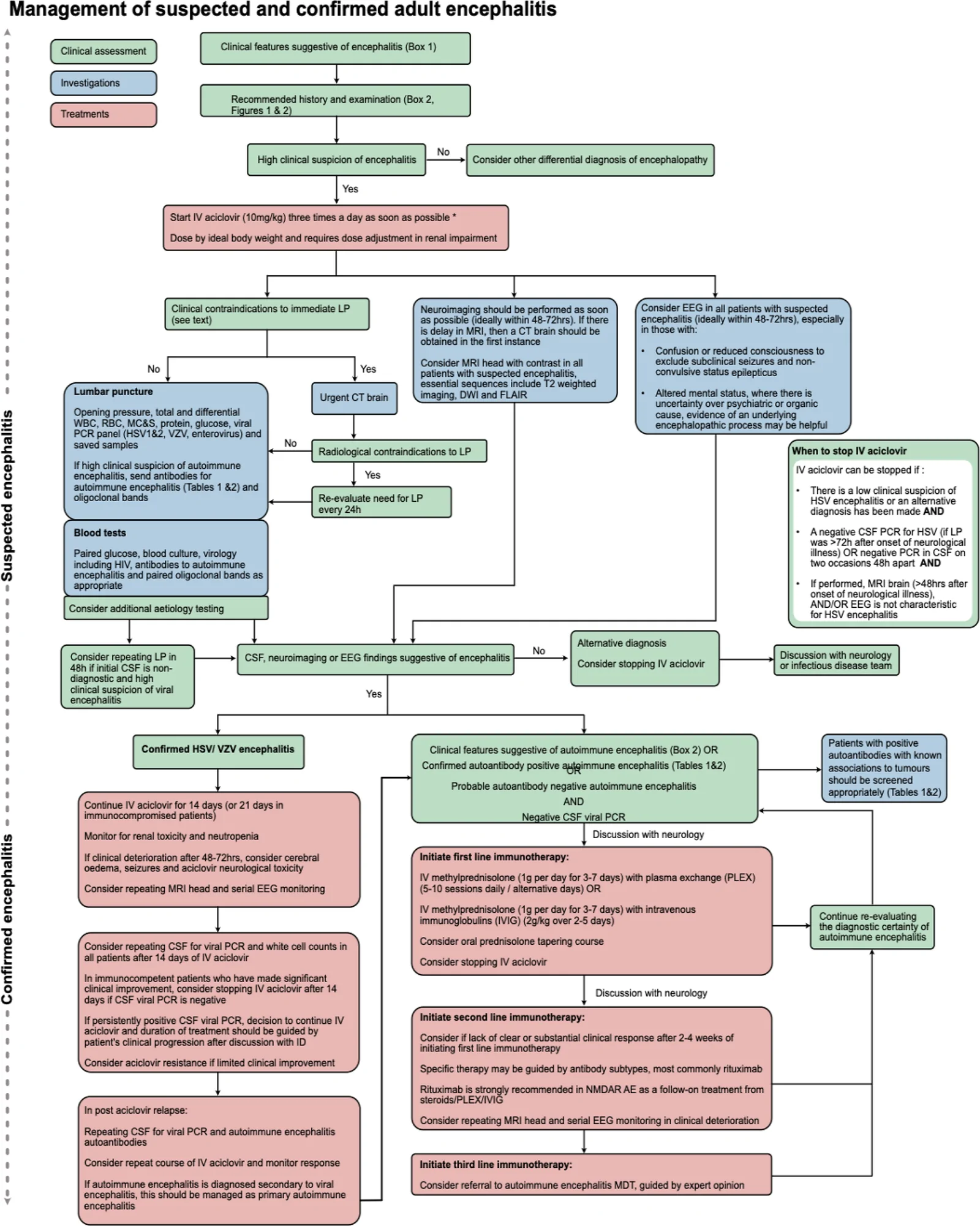
An algorithm for investigating and managing adults with suspected and confirmed encephalitis. *Dose may need increasing to 15mg/Kg if VZV is confirmed on PCR. AE, Autoimmune Encephalitis; CSF, cerebrospinal fluid; DWI, Diffusion Weighted Imaging; EEG, electroencephalogram; HSV, herpes simplex virus; LP, lumbar puncture; MC&S, Microscopy, Culture & Sensitivity; MDT, multidisciplinary team; NMDAR, N-methyl D aspartate receptor; RBC, Red Blood Cells; VZV, varicella zoster virus; WBC, white blood cell.

## Treatment of infectious encephalitis

Most patients with suspected encephalitis nonetheless require empirical treatment with 10 mg/kg of intravenous aciclovir 8 hourly, which should be started as soon as possible, ideally within 6 hours of admission, and certainly within 24 hours of admission.^51^ As aciclovir can rarely cause crystal nephropathy, the dose should be adjusted in those with impaired renal function. Aciclovir is continued when the PCR is positive for HSV-1, HSV-2 or VZV as it substantially reduces mortality.^64 65^ With a positive VZV PCR, there have been suggestions to increase the dose of aciclovir to 15 mg/kg 8 hourly based on in vitro experiments, although there is insufficient evidence to direct clinical care.^66^ Aciclovir should be continued for at least 14 days in the immunocompetent and 21 days in the immunocompromised, and many advise obtaining a repeat PCR at the end of the treatment course, particularly when there is limited clinical improvement or in the immunocompromised patient.^6^ In a recent randomised controlled trial of adjunctive dexamethasone, 17% of patients were positive for HSV in CSF at 14 days and there was no significant difference between dexamethasone and placebo groups.^67^ However, a positive CSF PCR does not necessarily imply active viral replication at this stage of infection. Nevertheless, given the relatively low risk of continued aciclovir and the high risk of ongoing viral infection causing progressive brain injury, the balance of risk–benefit often favours continuing aciclovir until CSF PCR is negative. Aciclovir should, however, be stopped when making an alternative diagnosis or when the PCR is positive for other viruses, such as enterovirus, West Nile virus or Eastern equine virus, for which aciclovir is ineffective.^66 68^ Other organisms are tested based on epidemiological risk or clinical suspicion and treated accordingly. For example, ganciclovir, foscarnet or cidofovir is considered for CMV or HHV-6B encephalitis, oseltamivir for influenza and ribavirin for measles.^6^

Supportive treatment of encephalitis can be challenging, and patients often have long inpatient stays. Seizures can be refractory, and scoring systems have been developed to stratify seizure risk, highlighting the importance of HSV and autoimmune encephalitis. However, we need prospective studies to determine the role of primary seizure prophylaxis and there is limited evidence to suggest particular antiseizure medicines.^69 70^ In patients with significant brain swelling, corticosteroids may be used as well as osmotic diuretics when there is raised intracranial pressure, in addition to other conservative measures such as elevating the head to 30° When these measures fail, decompressive hemicraniectomy has been used in small numbers of cases.^4 6^

## Treatment of autoimmune encephalitis

If there is a suspicion of autoimmune encephalitis from onset based on clinical features, clinicians should consider first-line immune therapy, as evidence has shown that early time to immune therapy improves outcome.^71^ Giving immune therapy is further supported in those with negative viral PCR and cultures with positive autoantibodies and suggestive imaging.

The evidence for treatment strategies for autoimmune encephalitis derives from retrospective studies only, and by analogy with other antibody-mediated diseases; we await randomised controlled trials, but these are methodologically challenging. Current first-line treatment typically includes high-dose intravenous methylprednisolone, combined with or shortly followed by plasma exchange and/or intravenous immunoglobulin, guided by availability and clinical stability.^57 68 72 73^

For NMDAR-antibody encephalitis, many clinicians now favour giving second-line agents early, such as rituximab (although cyclophosphamide is occasionally used), as this can reduce relapse rate.^72 74 75^ Investigation for teratoma should be rapid, and tumours should be resected urgently, as this appears to markedly improve outcome and reduce risk of relapse. The response to initial therapy should be monitored closely, and where there is failure to improve, there is some experience in a small number of cases with tocilizumab (IL-6 antagonist) and bortezomib (proteasome inhibitor).^73^ LGI-1 antibody encephalitis tends to have a good response to first-line treatments, or even corticosteroids alone, and rituximab is likely to reduce the rate of relapse.^74^ Paraneoplastic encephalitis due to antibodies targeting intracellular epitopes (eg, anti-Ma, anti-Hu) often responds poorly to immune therapy (although this is usually given regardless and occasionally leads to improvement), and treatment relies largely on resecting the underlying malignancy.^74^

Treatment of antibody-negative autoimmune encephalitis, which is probably a heterogeneous entity encompassing different pathogenic mechanisms, is controversial. As above, consensus diagnostic criteria can provide support for treatment, but it is essential to exclude alternative causes (table 5 of differentials). Similar approaches to immune therapy tend to be employed as with antibody-positive cases, although in our experience, it is advisable to establish objective evidence of improvement following a trial of first-line treatment (ie, a composite measure of improvement on cognitive testing, EEG, MRI or seizure frequency) before progressing to more aggressive immunosuppressive treatments.

The use of maintenance immune therapy to prevent relapse in encephalitis associated with neuronal surface antibodies (table 3) selected seronegative cases often varies in practice.^57^ Nevertheless, the risk of relapse is between 12 and 35% for NMDAR-antibody and LGI-1-antibody encephalitides; patients who relapse require repeat courses of immune therapy.^76 77^ There is limited evidence on treatment duration, but 1–2 years is often considered acceptable.^57^ We need effective biomarkers to identify patients at risk of relapse who may benefit from maintenance treatment.

Neurological symptomatic relapse weeks after HSV encephalitis can occur due to persistent viral replication or a post-infectious autoimmune syndrome. Post-infectious autoimmune syndrome is mostly associated with positive antibodies to NMDAR, and possibly other cell surface neuronal and synaptic antigens.^78–80^ The clinical presentation may include abnormal movements such as choreoathetosis and behavioural or psychiatric symptoms overlapping with acute psychosis symptoms.^80^ Nevertheless, NMDAR antibodies may be detectable in serum in up to a third of patients following HSV encephalitis, and these do not necessarily indicate autoimmune encephalitis. Therefore, CSF NMDAR antibodies should be tested for and the results interpreted in the clinical context to distinguish between post-HSV encephalitis sequelae of acquired brain injury (eg, epilepsy and cognitive impairment) and true post-HSV encephalitis NMDAR-antibody encephalitis. However, we need more studies to identify those at risk of autoimmune relapse.

## Prognosis, outcome and rehabilitation

Mortality from encephalitis varies between 5% and 40%.^63 81 82^ Recovery is variable depending on the cause, severity and, crucially, the timing of starting treatment. Common sequelae include epilepsy, cognitive deficits, fatigue, emotional disturbances, behavioural and personality change.^7 83–85^ This may result in social effects such as loss of work, driving or education due to the acquired disability. Clinicians should also consider the impact on the mental health of the caregiver and the family.^7^ Subtle neurocognitive deficits and neuropsychiatric sequelae are increasingly recognised, particularly in autoimmune encephalitis, even when patients appear to have returned to normal life. They may score well on basic metrics such as the modified Rankin score, but this does not reflect the encephalitis patient outcomes and experience.^74 86^ Ideal patient follow-up would include tailored multidisciplinary rehabilitation by a team comprising a neurologist, occupational, speech and language, neuropsychological and neuropsychiatric therapy, although availability of these services varies. Patients and their caregivers should also be signposted early to organisations such as Encephalitis International for support and information (*www.encephalitis.info*).

## Conclusions

Encephalitis is a broad clinical entity, and favourable outcomes depend on timely intervention in both infectious and autoimmune cases. Recognising clinical phenotypes at presentation can help streamline investigations and differentiate encephalitis from common mimics such as septic encephalopathy and delirium. Research in encephalitis is rapidly evolving and ongoing across multiple domains, from diagnosis to treatment. Pathogen discovery using next-generation sequencing has a growing role, particularly in the immunocompromised and when performed on brain tissue, although results require cautious interpretation.

Beyond diagnostics, there are currently few evidence-based treatment options for viral encephalitis outside of acyclovir for HSV and VZV. We desperately need targeted immune therapy that decreases cerebral oedema and improves clinical outcomes. We also need future studies to identify those patients at risk of autoimmune relapse so that they could be targeted early.

Clinical trials are ongoing to ascertain the optimal therapy and duration of treatment for the various autoimmune encephalitides due to high variability in their response to immunosuppression, and these will inform evidence-based treatment.

Ultimately, treatment strategies are evolving for both infectious and autoimmune encephalitis and will be increasingly targeted, based on the likely cause and clinical/paraclinical predictors of outcome. Due to the relatively low incidence of encephalitis, the broad differential at the time of presentation and the range of potential causes, we need future trials with innovative design approaches, such as platform models. Potential patients should be identified early and—informed by clinical features, CSF and biomarker findings—stratified to both early and escalating immune therapies for both infectious and autoimmune causes.

**Key points**

- Encephalitis comprises a significant proportion of inpatient neurology workload.
- Clinicians should consider both infectious and autoimmune encephalitis simultaneously at presentation.
- Time to treatment is a key determinant of outcome in both infectious and autoimmune encephalitis.
- Recognising clinical syndromes in encephalitis can guide investigation and treatment.

**Further reading**

- Venkatesan A, Michael BD, Probasco JC, et al. Acute encephalitis in immunocompetent adults. Lancet. 2019;393(10172):702–16.
- Graus F, Titulaer MJ, Balu R, et al. A clinical approach to diagnosis of autoimmune encephalitis. Lancet Neurol. 2016;15(4):391–404.
- Encephalitis: global threats, trends and public health implications: a technical brief [Internet]. [cited 2025 Mar 5]. Available from: https://www.who.int/publications/i/item/9789240106475
- Granerod J, Huang Y, Davies NWS, Sequeira PC, Mwapasa V, Rupali P, et al. Global Landscape of Encephalitis: Key Priorities to Reduce Future Disease Burden. Clinical Infectious Diseases. 2023;77(11):1552–60.
- Alam AM, Easton A, Nicholson TR, Irani SR, Davies NWS, Solomon T, et al. Encephalitis: diagnosis, management and recent advances in the field of encephalitides. Postgraduate Medical Journal. 2023;99(1174):815–25.

## Data Availability

No data are available.
